# Increasing the performance of cucumber (*Cucumis sativus* L.) seedlings by LED illumination

**DOI:** 10.1038/s41598-022-04859-y

**Published:** 2022-01-17

**Authors:** Ali Mohamed Hamedalla, Muhammad Moaaz Ali, Waleed M. Ali, Mohamed A. A. Ahmed, Mohamed Omar Kaseb, Hazem M. Kalaji, Janina Gajc-Wolska, Ahmed Fathy Yousef

**Affiliations:** 1 College of Mechanical and Electronic Engineering, Fujian Agriculture and Forestry University, Fuzhou, 350002 China; 2 College of Horticulture, Fujian Agriculture and Forestry University, Fuzhou, 350002 China; 3 Department of Horticulture, College of Agriculture, University of Al-Azhar (Branch Assiut), Assiut, 71524 Egypt; 4 Plant Production Department (Horticulture-Medicinal and Aromatic Plants), Faculty of Agriculture (Saba Basha), Alexandria University, Alexandria, 21531 Egypt; 5 Horticulture Research Institute, Agriculture Research Center, Giza, 12119 Egypt; 6 Department of Plant Physiology, Institute of Biology, Warsaw University of Life Sciences SGGW, 159 Now-oursynowska 159, 02-776 Warsaw, Poland; 7 Institute of Technology and Life Sciences (ITP), Falenty, Al. Hrabska 3, 05-090 Raszyn, Poland; 8 Department of Vegetable and Medicinal Plants, Institute of Horticultural Sciences, Warsaw University of Life Sciences-SGGW, 166 Nowoursynowska Street, 02-787 Warsaw, Poland

**Keywords:** Environmental sciences, Environmental impact

## Abstract

Light is one of the most important limiting factors for photosynthesis and the production of plants, especially in the regions where natural environmental conditions do not provide sufficient sunlight, and there is a great dependence on artificial lighting to grow plants and produce food. The influence of light intensity, quality, and photoperiod on photosynthetic pigments content and some biochemical and growth traits of cucumber seedlings grown under controlled conditions was investigated. An orthogonal design based on a combination of different light irradiances, ratio of LEDs and photoperiods was used. Treaments consisted of three light irradiance regimes (80, 100, and 150 µmol m^−2^ s^−1^) provided by light-emitting diodes (LEDs) of different ratios of red and blue (R:B) (30:70, 50:50, and 70:30) and three different photoperiods (10/14, 12/12, and 14/10 h). The white light was used as a control/reference. Plant height, hypocotyl length, stem diameter, leaf area, and soluble sugar content were highest when exposed to LM9 (150 µmol m^−2^ s^−1^; R70:B30; 12/12 h) light mode, while the lowest values for the above parameters were obtained under LM1 (80 µmol m^−2^ s^−1^; R30:B70; 10/14 h). Higher pigments contents (chlorophyll *a*, chlorophyll *b*, and carotenoid) were obtained when light regime LM9 (150 µmol m^−2^ s^−1^; R70:B30; 12/12 h) was applied. In general, cucumber seedlings grown under the LM9 regime showed a significant increase in growth as well as photosynthetic capacity. It seems that the content of photosynthetic pigments is the key factor responsible for the performance of cucumber seedlings grown under different lighting modes, compared to other traits studied. We recommend monitoring the content of chlorophyll *a, b*, and their ratio value when studying the light requirement of cucumber plants.

## Introduction

The cucumber is an economically important vegetable grown in over 80 countries worldwide^1^, and its annual production is estimated to be about 80 million tons, including about 3 million tons grown in the European Union (EU 28)^2^. High temperatures, humidity, light irradiance, and nutrient availability are ideal conditions for this typical subtropical plant, which is highly sensitive to adverse environmental conditions^3^. Characterized by their tenderness, these plants thrive in a temperature range between 18.3 to 23.9 °C, with a minimum temperature of 15.6 °C and a maximum of 32.2 °C. Germination of the cucumber takes place in soil that has a temperature range from 15.6 to 35 °C. However, germination is substantially impeded below 15.6 °C^4^.

The energy given by light is a key concern in cucumber cultivation, as it is in other plant development, and temperature regulation must be addressed in conjunction with light irradiance^5^. The total plant leaf area, carbohydrate production, and, consequently, productivity are all affected by radiation^6^. During the winter months, low carbohydrates supply and reduced output may lead many plants to fail^7^. As a result, the quality of the vegetable is directly influenced by light. The crops cultivated in such low light levels have less dry matter, and the colour is green when harvested but quickly becomes yellow when stored. The sensitivity of these young vegetables to low light irradiance is greater than that of the older vegetables from the same plant^3^.

The use of artificial lighting suitable for indoor cultivation under controlled environments has increased plant productivity in densely populated areas and space missions^8^. The spectral characteristics of electric light sources must meet the physiological requirements of plants for photosynthesis and photomorphogenic development^9^. However, the distribution and variation of the spectrum of conventional light sources (fluorescent tubes, sodium vapor lamps, metal halides etc.) is fixed and may not be ideal for the light requirements of different plant species. Light-emitting diodes (LEDs) are on the rise and have great potential for horticultural lighting due to their energy efficiency, longevity, and flexibility of use^10^. In addition, LEDs are becoming more suitable for research and commercial agriculture under controlled conditions due to their low radiation, heat, and broad spectral match^11^.

Brown et al.^12^ and Tennessen et al.^13^ reported that plant light demand research can determine the various functions of light properties in terms of spectral irradiance when growing conditions have been supplied with LEDs and modified to emit light photons at specific wavelengths. It is worth noting that LED provides the ideal spectral distribution range that promotes plant growth with optimal longevity and light energy efficiency^14^. During the shift from conventional to LED light sources, many inventions have been made, such as the combination of fluorescent lamps and LED^15^, the replacement of fluorescent tubes with LEDs^16^, and the retrofitting of LEDs for fluorescent tubes without ballasts^17^.

Many works have attempted to explain the influence of light on growth, development, morphology, and photosynthesis in various plants^18–21^. The combination of LED light quality has been observed to strongly influence the physiological and developmental processes of plants^22–26^.

Many scholars have reported different accounts of plants grown under different illumination irradiances, providing insights into plant growth and development as well as their photoperiodic requirements^25,27–30^.

The aim of this study was to determine the best light irradiance, light quality, and photoperiod for the growth of cucumber (*Cucumis sativus* var. Building No. 4) seedlings and to find out which physiological characteristic is the best as an indicator of optimal lighting. This experiment was designed on the hypothesis that the response of the photosynthetic apparatus of cucumber seedlings and their growth parameters will be varied base on the characteristics of the applied light modes.

## Results

### Growth parameters

**Figure 1:**
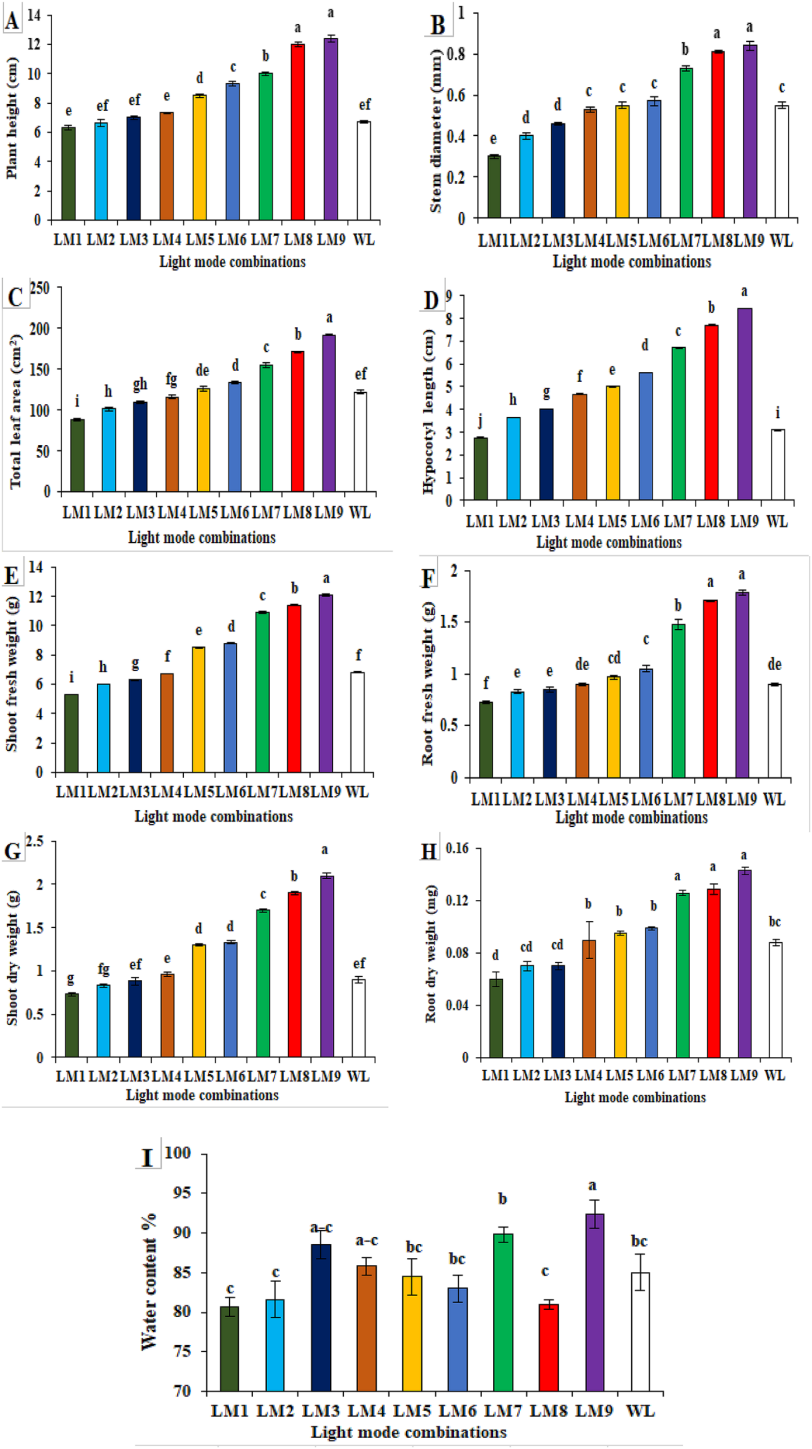
Effect of modes LED light on plant morphology and growth characteristics of cucumber seedlings; where Plant height (**A**), Stem diameter (**B**), Total leaf area (**C**), Hypocotyl length (**D**), Shoot Fresh weight (**E**), Root Fresh weight (**F**), Shoot dry weight (**G**), Root Dry weight (**H**), Water content % (**I**). Means followed by the same letter within the same series are not significantly different according to Duncan’s multiple range test (*P* ≤ 0.05). See Table 4 for LM abbreviations.

The effect of light type LED on plant morphology and growth characteristics of cucumber seedlings is shown in Fig. 1. Different light conditions had significant effects on the morphological characteristics of cucumber seedlings. Plant height, stem diameter, total leaf area, hypocotyl length, shoot fresh weight, root fresh weight, shoot dry weight and root dry weight were significantly higher under LM9 treatment than the other treatments, while the lowest value for all these parameters was observed under LM1. Plant height, stem diameter, total leaf area, hypocotyl length, shoot fresh weight, root fresh weight, shoot dry weight and root dry weight were increased by 85.07, 52.73, 57.37, 172.81, 77.94, 98.88, 133.33 and 62.5% when LM9 was applied on cucumber seedlings (Fig. 1A–H) as compared to control (WL) while application of LM8 increased these attributes by 79.1, 47.27, 40.16, 149.19, 67.64, 90, 111 and 46.59% respectively as compared to control (WL). The water content of plants exposed to LM9 (92.4%) was significantly higher than under the other LED light modes, although the values were not significantly different between LM1–LM6, LM8, and WL (Fig. 1I).

On the other hand, according to the R-values, the order of influence of the three factors on growth characteristics of cucumber seedlings was observed in this study by using the orthogonal array design (Table 1). Table 1 shows that the order of impact of the three factors on plant height, stem diameter, leaf area, Hypocotyl length, shoot fresh weight, root fresh weight, shoot dry weight, root dry weight, and water content was (A > B > C), (A > B > C), (A > B > C), (A > B > C), (A > B > C), (A > B > C), (A > B > C), (A > C > B), and (C > B > A), respectively.

**Table 1:** Results of the range and ANOVA of the L9 (3^3^) matrix for the influence of combined, irradiances of LEDs light (Factor A), light spectral ratios (Factor B), and photoperiod (Factor C) on growth characteristics of cucumber seedlings.

		Plant height (cm)		Stem diameter (mm)		Leaf area (cm^2^)		Hypocotyl length		Fresh weight				Dry weight				Water content %	
										Shoot (g)		Root (g.)		Shoot (g)		Root (g)			
Factors	Value	*R*	*P*	*R*	*P*	*R*	*P*	*R*	*P*	*R*	*P*	*R*	*P*	*R*	*P*	*R*	*P*	*R*	*P*
	A	4.83	< 0.0001	0.407	< 0.0001	73.33	< 0.0001	4.15	< 0.0001	5.60	< 0.0001	0.860	< 0.0001	1.09	< 0.0001	0.066	< 0.0001	4.13	< 0.0001
	B	1.70	0.0637	0.103	0.0012	25.00	0.0081	1.30	0.0048	1.43	< 0.0001	0.197	0.0644	0.31	0.0060	0.008	0.4585	5.60	< 0.0001
	C	0.70	0.9069	0.030	0.0225	6.33	0.0496	0.34	0.0782	0.30	0.9957	0.073	0.2504	0.03	0.7645	0.009	0.0134	6.03	< 0.0001
ELF		A > B > C		A > B > C		A > B > C		A > B > C		A > B > C		A > B > C		A > B > C		A > C > B		C > B > A	
BCm		A3B3C2		A3B3C2		A3B3C2		A3B3C2		A3B3C2		A3B3C2		A3B3C2		A3B3C2		A3B3C2	

Based on the average of growth characteristics derived from three factors at each level, the best combination of different factors with the levels to get the highest results on growth performance was A_3_B_3_C_2_, which indicated that the maximum of these parameters presented at the irradiance of light (150 µmol m^−2^ s^−1^), the ratio of (R70:B30), and photoperiod (12/12 h).

ANOVA (Table 1) showed that these three factors were significant effects on growth performance parameters of cucumber seedlings (*p* ˂ 0.05), excepted factor C on plant height, hypocotyl length, shoot fresh weight, root fresh weight, and shoot dry weight had no significant effects, and also excepted factor B on root fresh weight and root dry weight had no significant effects.

### Photosynthetic pigments content

**Figure 2:**
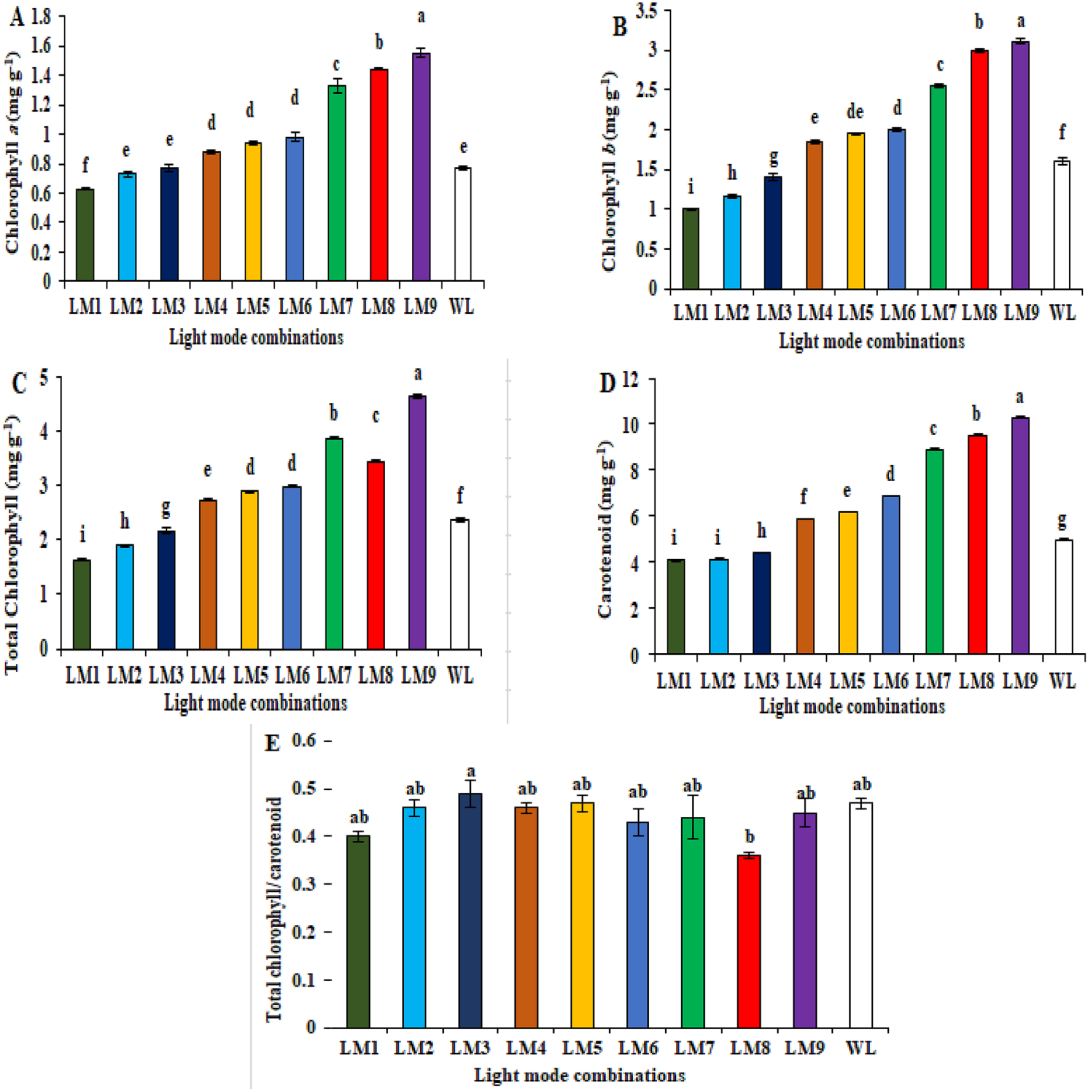
Effect of modes LED light on photosynthetic pigments content [chlorophyll *a* (**A**), chlorophyll *b* (**B**), total chlorophyll (**C**), carotenoid (**D**), and total chlorophyll/carotenoid contents (**E**)] in cucumber seedlings. Means followed by the same letter within the same series are not significantly different according to Duncan’s multiple range test (*P* ≤ 0.05). See Table 4 for LM abbreviations.

The contents of chlorophyll and carotenoids in the leaves of cucumber seedlings under different LED light modes is shown in Fig. 2. Compared with WL treatment, the levels of Chl *a*, Chl *b*, total chlorophyll and carotenoid in the leaves of cucumber seedlings were higher with LM9 than with the other modes of LED light, with LM7 and LM8 showing no statistical difference, while LM1 mode showed the lowest levels of Chl *a*, Chl *b* and carotenoid (Fig. 2A–D). The ratio of carotenoid to total chlorophyll was higher with LM3 than the other modes of LED light, with LM4, LM5 and WL showing no statistical difference between the values, while LM1 mode showed the lowest ratio (Fig. 2E).

On the other hand, according to the R-values, the order of influence of the three factors on photosynthetic pigments content of cucumber seedlings was observed in this study (Table 2). Table 2 shows that the order of impact of the three factors on chlorophyll a, chlorophyll b, total chlorophyll, carotenoid, and total chlorophyll/carotenoid was (A > B > C), (A > B > C), (A > B > C), (A > B > C), and (C > A > B), respectively.

**Table 2:** Results of the range and ANOVA of the L9 (3^3^) matrix for the influence of combined, irradiances of LEDs light (Factor A), light spectral ratios (Factor B), and photoperiod (Factor C) on photosynthetic pigments content and biochemical traits of cucumber seedlings.

		Chlorophyll a		Chlorophyll b		Total chlorophyll		Carotenoid		Total chlorophyll/ carotenoid		Nitrate content		Soluble protein		Soluble sugar	
Factors	Value	*R*	*P*	*R*	*P*	*R*	*P*	*R*	*P*	*R*	*P*	*R*	*P*	*R*	*P*	*R*	*P*
	A	0.73	< 0.0001	1.70	< 0.0001	2.09	< 0.0001	5.36	< 0.0001	0.037	0.0130	630.00	< 0.0001	5.68	< 0.0001	3.32	< 0.0001
	B	0.15	0.0078	0.37	0.0826	0.53	< 0.0001	0.89	0.0551	0.027	0.0063	193.33	< 0.0001	1.25	< 0.0001	0.68	0.0131
	C	0.04	0.0504	0.08	0.1458	0.41	< 0.0001	0.32	0.4363	0.070	0.0004	63.33	0.0404	0.26	0.0336	0.14	0.8695
ELF		A > B > C		A > B > C		A > B > C		A > B > C		C > A > B		A > B > C		A > B > C		A > B > C	
BCm		A3B3C2		A3B3C2		A3B3C2		A3B3C2		A1B3C3		A1B3C3		A1B3C3		A3B3C2	

Where: Range value (R), the range of difference between the maximum and minimum average; ELF, The most influential level factors on the parameter gradually; BCm, The best level combination for each parameter; (*P*-value), ANOVA analysis of variance.

Based on the average of photosynthetic pigments content derived from three factors at each level, the A_3_B_3_C_2_ was the best combinations gave the highest chlorophyll a, chlorophyll b, total chlorophyll, and carotenoid, which indicated that the maximum of these parameters presented at (irradiance 150 µmol m^−2^ s^−1^ + ratio (R70:B30) + photoperiod 12/12 h). While the best combination of different factors with the levels for the highest total chlorophyll/carotenoid was A_1_B_3_C_3_, which indicated that the maximum of these parameters presented with (irradiance 80 µmol m^−2^ s^−1^ + ratio (R70:B30) + photoperiod 14/10 h).

ANOVA (Table 2) showed that these three factors were significant effects on photosynthetic pigments content of cucumber seedlings (*p *˂ 0.05), excepted factor B on chlorophyll b, and factor C on chlorophyll b and Carotenoid had no significant effects.

### Biochemical traits

**Figure 3:**
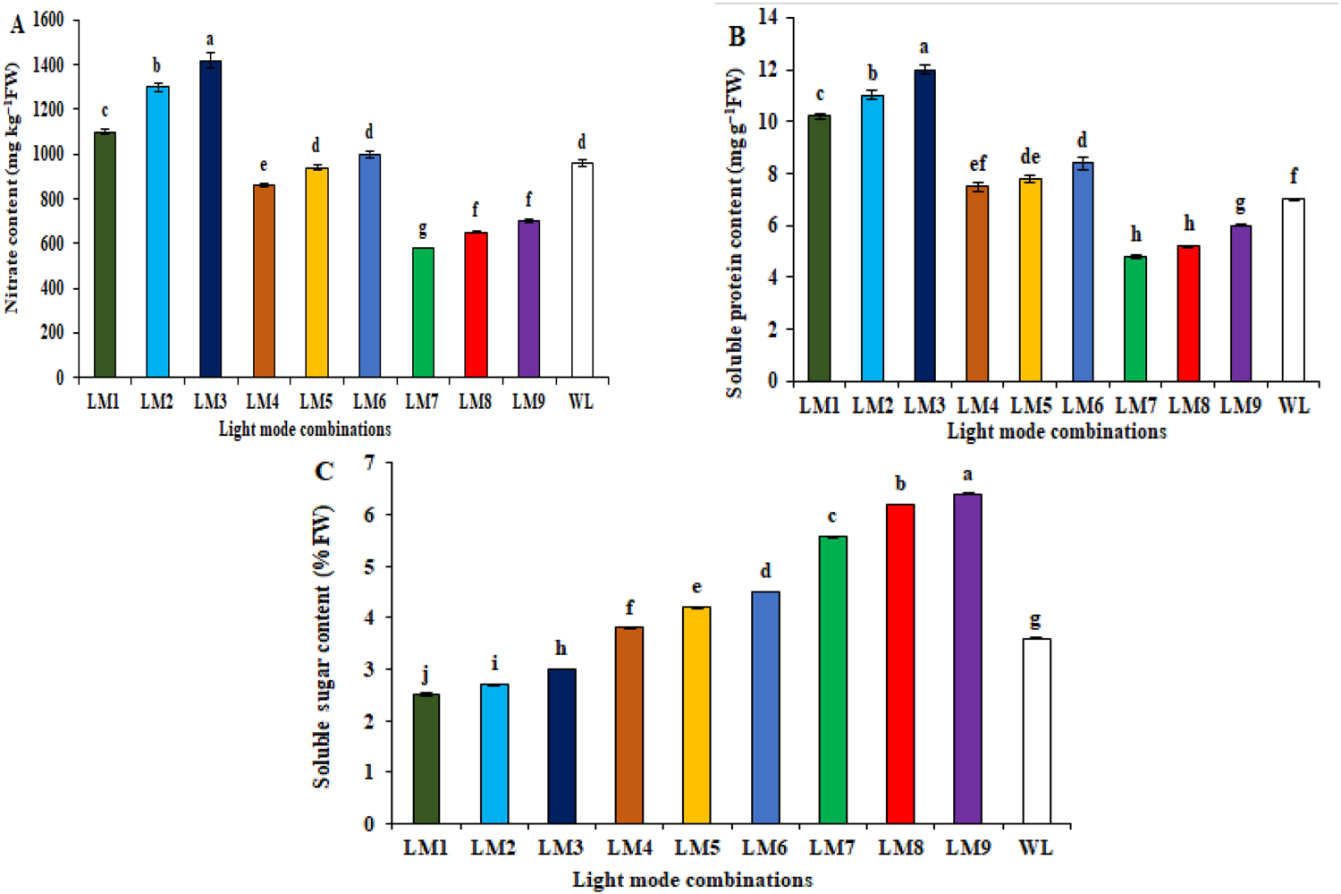
Effect of modes LED light on biochemical traits; Nitrate content (**A**), Soluble protein content (**B**), and Soluble sugar content (**C**) in cucumber seedlings. Means followed by the same letter within the same series are not significantly different according to Duncan’s multiple range test (*P* ≤ 0.05). See Table 4 for LM abbreviations.

Light modes of LEDs had significant effects on the accumulation of biochemical compounds, including the contents of nitrate, soluble protein, and soluble sugar (Fig. 3). LM3 provided the highest nitrate and soluble protein contents; this shows the importance of red light in improving nitrate and soluble protein content (Fig. 3A,B). Also, LM9 exhibited the highest accumulation of soluble sugar in the cucumber seedlings (6.40%FW) (Fig. 3C).

On the other hand, according to the *R*-values, the order of influence of the three factors on biochemical traits of cucumber seedlings was observed in this study (Table 2). Table 2 shows that the order of impact of the three factors on nitrate content, soluble protein, and soluble sugar contents was (A > B > C) with all of them.

Based on the average of biochemical traits derived from three factors at each level, the best combination of different factors with the levels for the highest nitrate content and soluble protein was A_1_B_3_C_3_, which indicated that the maximum of these parameters presented at (irradiance 80 µmol m^−2^ s^−1^ + ratio (R70:B30) + photoperiod 14/10 h). While the best combination of different factors with the levels for the highest soluble sugar content was A_3_B_3_C_2_, which indicated that the maximum of soluble sugar content presented with (irradiance 150 µmol m^−2^ s^−1^ + ratio (R70:B30) + photoperiod12/12 h).

ANOVA (Table 2) showed that these three factors were significant effects on biochemical traits of cucumber seedlings (*p ˂ *0.05), excepted factors C on soluble sugar content had no significant effects.

### Correlation analysis

Pearson’s correlation^31^ was carried out among the morphological, photosynthetic pigments content, and biochemical traits observed in this study as shown in Table 3. There was a highly significant positive correlation between PH with [(SD R^2^ = 0.935), (TLA R^2^ = 0.965), (HL R^2^ = 0.983), (SFW R^2^ = 0.977), (RFW R^2^ = 0.970), (SDW R^2^ = 0.990), (RDW R^2^ = 0.949), (Chl *a* R^2^ = 0.977), (Chl *b* R^2^ = 0.969), (ToCh R^2^ = 0.916), (Car. R^2^ = 0.975), and (Sug. R^2^ = 0.972)]. While the correlation relation between PH with (WC%, ToCh/Car., Nit., and Pro.) were found significantly negative (R^2^ = − 0.866, − 0.458, − 0.753, and − 0.734, respectively). Additionally, a positive significant relationship was found between SD and [(TLA R^2^ = 0.983), (HL R^2^ = 0.937), (SFW R^2^ = 0.963), (RFW R^2^ = 0.956), (SDW R^2^ = 0.951), (RDW R^2^ = 0.984), (Chl *a* R^2^ = 0.966), (Chl *b* R^2^ = 0.986), (ToCh R^2^ = 0.943), (Car. R^2^ = 0.962), and (Sug. R^2^ = 0.981)]. Whereas the negative correlations were found between SD and WC %, Nit., and Pro. (R^2^ = − 0.953, − 0.814, and − 0.846, respectively). The relationship between Nit. and Pro. with all morphological traits was high significantly negative, while the relationship between Sug. and all morphological traits was high significantly positive as shown in Table 3.

**Table 3:** Correlation coefficient evaluation between studied traits in cucumber seedlings.

	PH	SD	TLA	HL	SFW	RFW	SDW	RDW	WC%	Chl *a*	Chl *b*	ToCh	Car.	ToCh/Car.	Nit.	Pro.	Sug.
PH	1																
SD	0.935**	1															
TLA	0.965**	0.983**	1														
HL	0.983**	0.937**	0.957**	1													
SFW	0.977**	0.963**	0.974**	0.969**	1												
RFW	0.970**	0.956**	0.974**	0.960**	0.968**	1											
SDW	0.990**	0.951**	0.976**	0.981**	0.994**	0.976**	1										
RDW	0.949**	0.984**	0.984**	0.953**	0.981**	0.962**	0.973**	1									
WC%	− 0.866**	− 0.953**	− 0.917**	− 0.869**	− 0.919**	− 0.846**	− 0.887**	− 0.942**	1								
Chl *a*	0.977**	0.966**	0.979**	0.983**	0.984**	0.991**	0.989**	0.980**	− 0.881**	1							
Chl *b*	0.969**	0.986**	0.982**	0.971**	0.977**	0.966**	0.976**	0.987**	− 0.934**	0.983**	1						
ToCh	0.916**	0.943**	0.960**	0.942**	0.952**	0.917**	0.950**	0.973**	− 0.907**	0.953**	0.952**	1					
Car.	0.975**	0.962**	0.973**	0.976**	0.986**	0.974**	0.987**	0.987**	− 0.904**	0.991**	0.987**	0.960**	1				
ToCh/Car.	− 0.458	− 0.279	− 0.297	− 0.366	− 0.368	− 0.436	− 0.39	− 0.313	0.195	− 0.384	− 0.363	− 0.158	− 0.415	1			
Nit.	− 0.753*	− 0.814**	− 0.781**	− 0.747*	− 0.813**	− 0.786**	− 0.792**	− 0.865**	0.806**	− 0.811**	− 0.839**	− 0.805**	− 0.859**	0.466	1		
Pro.	− 0.734*	− 0.846**	− 0.795**	− 0.720*	− 0.811**	− 0.777**	− 0.778**	− 0.872**	0.858**	− 0.796**	− 0.840**	− 0.796**	− 0.840**	0.389	0.980**	1	
Sug.	0.972**	0.981**	0.981**	0.966**	0.988**	0.967**	0.983**	0.992**	− 0.940**	0.984**	0.995**	0.953**	0.993**	− 0.392	− 0.857**	− 0.858**	1

*Correlation is significant at the *P* ≤ 0.05 level.

**Correlation is significant at the *P* ≤ 0.01 level, by using Pearson correlation coefficients. Where *PH* Plant height, *SD* Stem diameter, *TLA* Total leave area, *HL* Hypocotyl length, *SFW* Shoot fresh weight, *RFW* Root fresh weight, *SDW* Shoot dry weight, *RDW* Root dry weight, *WC%* Water content, *Chl a* Chlorophyll *a*, *Chl b* Chlorophyll *b*, *ToCh* Total Chlorophyll contents, *Car.* Carotenoid, *ToCh/Car.* Total Chlorophyll contents/Carotenoid, *Nit.* Nitrate content, *Pro.* Protein content, and *Sug.* Sugar content.

## Discussion

For proper growth and development, plants are grown under constantly changing light conditions. Some light wavelengths are critical for plant growth and development. Plants are observed to detect subtle changes in light quality by light receptors. These light receptors can initiate signal transduction through various pathways to alter plant appearance^32–34^. It has been observed that photosynthetically active radiation (PAR; 400–700 nm) plays a direct role in photosynthetic processes of plants. Red light in the range of 610–700 nm and blue at 425–490 nm is the optimal light spectrum for photosynthesis in plants^35^. Recently, there are many research results that focused on the effects of LED light on morphogenesis and photosynthesis. They observed that red and blue light improved plant production when light irradiance and quality were controlled^36–38^.

### Growth parameters

The photosynthetic process in leaves requires the capture of light, which is influenced by the wavelength (light spectrum), intensity and angle of incidence^39^, and total leaf area. It was observed that cucumber seedlings responded strongly to LM9 in terms of plant height, stem diameter, total leaf area, hypocotyl length, shoot fresh weight, root fresh weight, shoot dry weight, and root dry weight (Fig. 1). This finding agreed with the results of Naznin et al.^37^ and Yang et al.^40^ in pepper seedlings and Yang et al.^41^ in tomato seedlings, they observed that a mixture of red and blue LED light was efficient in producing strong seedlings. The combination of red and blue light was shown to be the most beneficial in promoting plant growth and development in the majority of research. When cucumber seedlings were grown under a combination of red and blue light (R5: B5), yields were higher than when they were grown under red light only^42^. Under a combination of blue and red light, Kim and Hwang confirmed that high grade 'Mini Chal' tomato (*Solanum lycopersicum* L.) could be grown in plant factories^43^. Furthermore, the barrier tissue cells in the leaves were very well developed and the spongy tissue cells were organized in an ordered manner under red + blue light^44^.

Combining red and blue light is more effective for plant development than monochromatic red light. Phytochrome-dependent elongation of hypocotyls and cotyledons was seen in plants cultivated under monochromatic red light^45^. Plants exposed to a mixture of red and blue light had greater photoreceptor excitation, like phytochromes, cryptochromes, and phototropins, and had higher photosynthetic activity than plants exposed to monochromatic red or blue light^46^. According to Yousef and coauthors showed that a combination of R and B LED light with a high R portion was effective in producing vigorous grafted tomato seedlings compared to blue and red light alone^20,47,48^. Also, Dong et al.^49^, as compared to blue light alone, red light alone, and sunshine, combined red and blue light (1/3 blue light at 450–460 nm + 2/3 red light at 620–630 nm, at 400 lx and 12 h photoperiod for 60 days) improved the DW and bio efficiency of *Cordyceps militaris* mushroom. Plant production was considerably improved for most species when combined light wavelengths with a considerable proportion of red light supplemented by blue light were used^50^.

### Effects of mode (regime) on photosynthetic pigments content

Chlorophyll content directly affects photosynthetic ability and primary production^51^. Moreover, the chlorophyll content of plants is affected by the quality of light. Many studies have explained the beneficial effects of using blue lights^21,38,41^. Our results showed that the combination of red and blue LED light with high red light (LM9) was observed to be favorable for chlorophyll a, chlorophyll b, and carotenoids content (Fig. 2). This is in agreement with the findings of Yang et al.^41^ on tomato seedlings and on pepper seedlings^37^, they have observed that the content of these pigments was more when seedlings were exposed to a mixture of red and blue LED lights than white fluorescent lights exposure.

Chlorophyll content, photosynthetic enzyme activity, stomatal aperture and carbohydrate release in plants were all affected by red and/or blue light^52^. Because of the high amount of carbohydrates in the leaves, red light increased the total chlorophyll content in plants, which encouraged photosynthesis. However, it prevented the movement of carbohydrates from the leaves to enhance photosynthesis, suppressing photosynthesis^52^. By raising the ratio of chl a/b, enhancing the activities of ribulose-1,5-bisphosphate carboxylase (Rubisco) and phosphoenolpyruvate carboxylase, and encouraging stomatal opening, blue light improved photosynthesis per unit leaf area^53^. Plant yield was affected by red and/or blue light during morphogenesis. Cell division and expansion were aided by red light, resulting in increased leaf area and root elongation, whereas cell division and expansion were hindered by blue light, resulting in reduced leaf area and root elongation^54^. Plant photomorphogenesis was affected by blue light, which increased chlorophyll a/b ratios and facilitated stomatal opening^55^. Reduced photon uptake due to reduced leaf area could be the cause of reduced plant growth under LM1.

### Effect of light mode (regime) on chosen biochemical traits

The study presented the significance of the use of red light in metabolite accumulation in cucumber seedlings. LM3 was more effective in increasing nitrate and soluble sugar content in cucumber seedlings while WL was observed to increase soluble protein content. The study by Bian et al.^56^ proved that soluble sugar in lettuce was higher when irradiated with red, green, and blue LED light (4:1:1) than under other types of LED light. Also, Xiaoying et al.^18^ observed that tomato seedlings grown under blue LED light had higher soluble sugar levels than under other types of LED light^57^, they also observed that soluble sugar levels were higher than other types of LED light in pepper, tomato and cucumber seedlings grown under red LED light and (red:blue). Their results proved that soluble sugars and proteins respond to different light qualities in vegetable crops grown under controlled conditions and that this also varies among different species and cultivars. In the present study, it was found that at a light irradiance of (80 ± 2 μmol m^−2^ s^−1^) and 14/10 h photoperiods, a combination of R70:B30 LED light was more effective in reducing nitrate concentration for cucumber seedlings than the white fluorescent light. Bian et al.^56^ reported that the effect of red and blue light had negative effects on nitrate assimilation by decreasing the activity and expression of nitrate assimilation-related genes NR and NiR in hydroponically grown lettuce, while the addition of green light with red and blue light had positive effects on nitrate assimilation by increasing activity.

**Figure 4:**
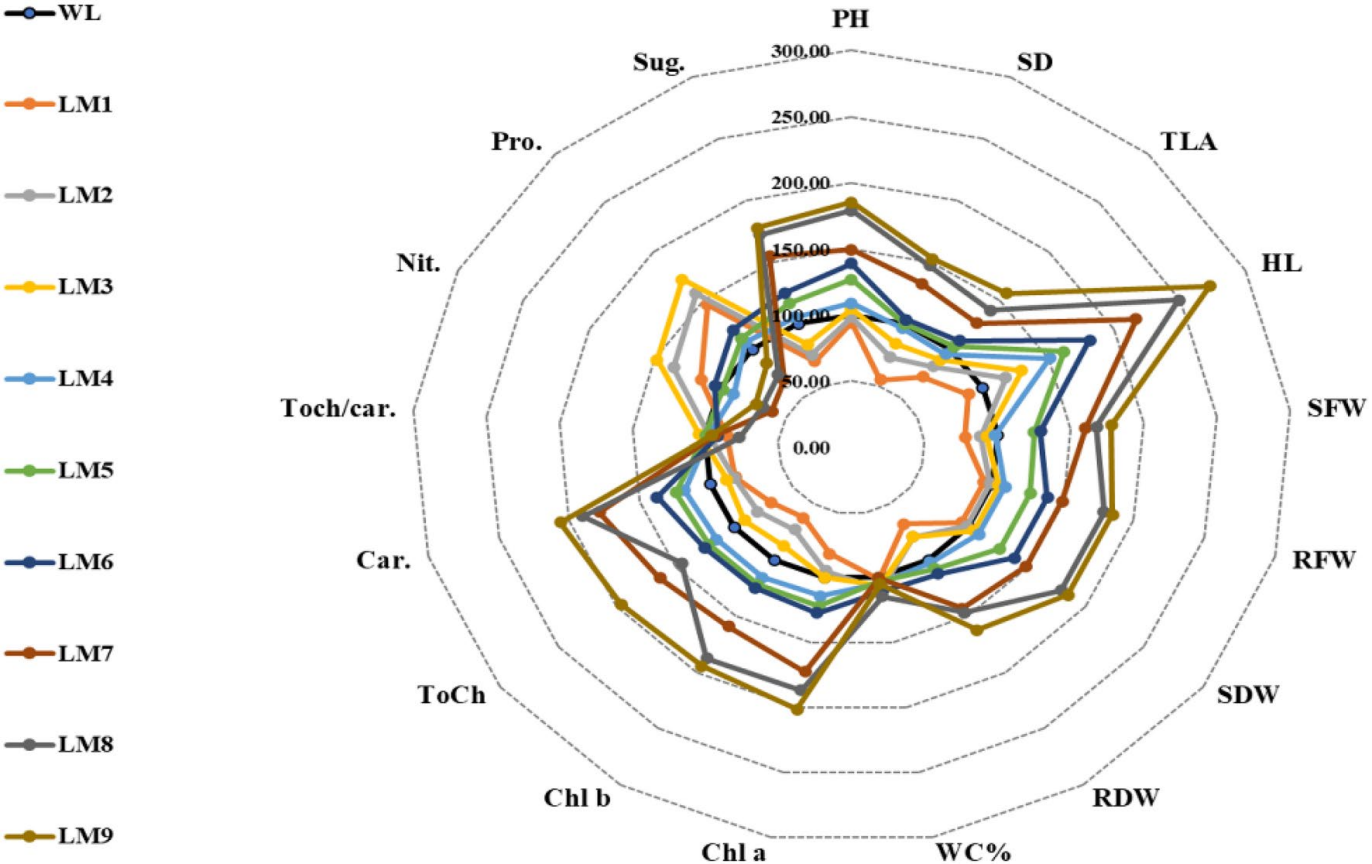
Radar plot of all studied traits under various light modes. Where *PH* Plant height, *SD* Stem diameter, *TLA* Total leave area, *HL* Hypocotyl length, *SFW* Shoot fresh weight, *RFW* Root fresh weight, *SDW* Shoot dry weight, *RDW* Root dry weight, *WC %* Water content, *Chl a* Chlorophyll a, *Chl b* Chlorophyll b, *ToCh* Total Chlorophyll contents, *Car.* Carotenoid, *ToCh/Car.* Total Chlorophyll contents/Carotenoid, *Nit.* Nitrate content, *Pro.* Protein content, and *Sug.* Sugar content.

The correlation analysis performed showed that most of the tested parameters were significantly and positively correlated. Only traits such as water content %, nitrate, and protein content were negatively associated. Total chlorophyll/carotenoid ratio and did not show significant relationship with the other traits studied (Fig. 4).

## Materials and methods

### Growth conditions and plant materials

**Figure 5:**
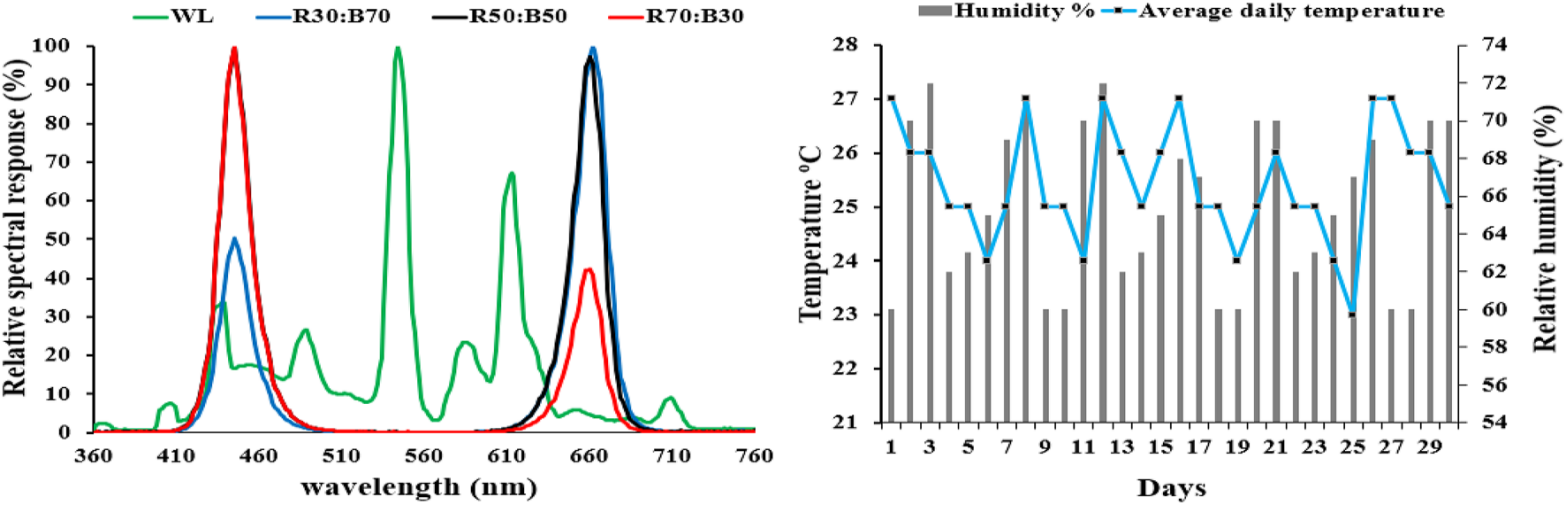
Spectrum distribution of the treatments LED light and in the experiment and environmental conditions.

The experimental system included 10 chambers; each had a dimension of 60 × 60 × 60 cm. The details of growth conditions and LEDs light are shown in Table 4 and Fig. 5. The manufacturer of the tested LED lamps is Kedao Technology Corporation (Huizhou, China) with the type of UH-BLDT0510. The plant experiments were complied with local and national regulations and following Fujian Agriculture and Forestry University (Fujian, China) regulations. Cucumber seeds (*Cucumis sativus* var. Built No. 4) were provided by the Tianjin Kernel Cucumber Research Institute. Cucumber seeds were sown in 32-cell plug trays (W 4 cm × L 4 cm × H 6 cm/cell) that was filled with commercial growing substrate (N1: P1: K1 ≥ 3%, Organic matter ≥ 45% pH 5.5–6.5). Ten days after planting, the germinated seedlings were transferred to pots (W 10 cm × L 10 cm × H 8.5 cm) and were left there for 20 days. In total, 20 seedlings were sown in each growth chamber. Irrigation was provided for the seedlings daily or as required. Seedlings began receiving fertilization based on water-soluble nutrients two times per week through irrigation one week after sowing. The air conditioner and ventilation fans were relied on in the chambers to standardize temperatures as well as possible.

**Table 4:** Parameters of the LED light properties used in the study.

Modes	Photon flux density (μmol m^−2^ s^−1^)	Light spectral ratios Red: Blue	Photoperiod Light/Dark	Peak wave length λp (nm)	Layout of the L9 (3^3^) matrix		
					A	B	C
LM1	80 ± 2	R30:B70	10/14 h	660:460	1	1	1
LM2	80 ± 2	R50:B50	12/12 h	660:460	1	2	2
LM3	80 ± 2	R70:B30	14/10 h	660:460	1	3	3
LM4	100 ± 2	R30:B70	12/12 h	660:460	2	1	2
LM5	100 ± 2	R50:B50	14/10 h	660:460	2	2	3
LM6	100 ± 2	R70:B30	10/14 h	660:460	2	3	1
LM7	150 ± 2	R30:B70	14/10 h	660:460	3	1	3
LM8	150 ± 2	R50:B50	10/14 h	660:460	3	2	1
LM9	150 ± 2	R70:B30	12/12 h	660:460	3	3	2
WL	115 ± 2	–	12/12 h	544	–	–	–

### Multiple-factor experiment design

The multiple-factor experimental regular fractional design L9 (3^3^) was used in this experiment (Table 4) i.e. 3 levels were chosen for each of the 3 improvement criteria and 9 tests from all possible combinations. When considering the technical feasibility of the advanced LED lighting unit, the parameters for improving the lighting system in the factory were chosen at the following levels:

A. The irradiances of LEDs light averaged over the whole time of plant growing period, PPFD (A1-A3): 80, 100, 150 μmol m^−2^ s^−1^.
B. The ratio of PPDD values from Red and Blue LEDs (B1-B3): (R:B) = 30:70, 50:50, and 70:30.
C. The light period during Light/Dark (C1–C3): 10/14 h, 12/12 h, and 14/10 h.

Additionally, a white fluorescent lamp (WL) was used as a control.

### Measurements and calculations

#### Measurement of growth and biomass parameters

On the 30th day after planting, growth metrics were measured. Using a ruler, the height of the plant was measured from the base of the rhizome to the top of the plant (cm). Digital calipers (mm) were used to measure stem diameter, and an electronic balance was used to weigh the fresh and dry mass (0.0001 g). Pandey and Singh's^58^ method for calculating total leaf area (cm^2^) was used. To acquire the dry weight, fresh shoots and roots were placed in Petri plates without cover and placed in a drying oven at 75 °C for at least 48 h.

#### Measurement of photosynthetic pigments

After 30 days of transplanting chlorophyll and carotenoid contents were determined from fresh medium-aged leaves with excluded the edges and veins of leaves. Tissues of fresh leaves (0.2 g) were cut, ground well then used in 5 mL of 95% ethanol and filtered, the filtrate was made up to 25 mL by adding 95% ethanol. Absorbance of the filtered solution at 665 nm (OD665), 649 nm (OD649) and 470 nm (OD470) was measured using a spectrophotometer (UV-5100B, Unico. Shanghai, China) while the chlorophyll content was determined using the equations below^59^: Chl a (mg g^−1^FW) = (13.95OD665 − 6.88OD649) V/200 W; Chl b (mg g^−1^FW) = (24.96OD649 − 7.32OD663) V/200 W; Total Chlorophyll (mg g^−1^FW) = Chl a + Chl b; C (mg g^−1^FW) = (1000OD470 − 2.05Chl a − 114.80Chl b) V/(245 × 200 W). Where (Chl a) = chlorophyll a, (Chl b) = chlorophyll b, (C) = carotenoid; (V) = volume (25 mL) and (W) = sample weight (g).

#### Measurement of biochemical traits

To measure the biochemical traits, fresh leaves were chopped into small pieces and fresh samples weighed (0.5 g, 0.2 g, and 0.5 g) for nitrate, protein, and sugar, respectively; they were used to determine the content of soluble nitrate^60^. The soluble protein content was evaluated using coomassie brilliant blue G250 method^61^. Also, the content of soluble sugar was evaluated using the anthrone colorimetric method^62^. The absorbance of the solution extracted was estimated at 410 nm (OD410), 595 nm (OD595), and 630 nm (OD630) using a UV-5100B spectrophotometer (Unico, Shanghai, China). The biochemical traits were expressed using the following equations: Soluble nitrate content (mg kg^−1^FW) = (C × VT)/(W × VS); Soluble protein content (mg g^−1^FW) = (C × VT)/(VS × W × 1000); Soluble sugar content (%) = (C/VS × VT)/(W × 106) × 100. Where C = nitrite; sugar (%) ; protein value from the standard curve, VT = total volume of samples extracted (mL), VS = taken sample solution (mL), and W = leaf fresh weight (g).

### Statistical analysis

All above mentioned measurements were made with 9 replicates. The Orthogonal Experimental design method was used to determine the number of experiments to be conducted. All the data were subjected to an one-way analysis of variance (ANOVA). Duncan's multiple range tests^63^ was used to test the significant difference between the means at 0.05 significance level using SPSS software (Version 16 SPSS Inc. Chicago, Illinois). The importance of the three factors for the measured parameters was assessed according to the effectiveness of each factor^64^ by the range value (*R*) using Excel 365 (v16.0). The most important impact factor has the greatest R-value. Correlation analysis was done, and Pearson correlation coefficients are shown^65^.

## Conclusions

Based on our experimental work and the data obtained, we propose the following mechanism or "cascade of effects": the light mode acts first on photosynthetic pigments. This, in turn, increases the photosynthetic output of plants and then the soluble sugar content, which could indicate a higher production of proteasomal. The latter were directly used by plants to build up the shoot and root biomass and increase the photosynthetic area of plants.

In summary, we believe that light irradiance is more important (has a greater effect) than light ratio and photoperiod in the case of cucumber seedlings (Fig. 4). This is evident in the case of LEDs of 150 μmol m^−2^ s^−1^ (LM 7, 8 and 9), where in general the highest values of the most studied traits/properties were observed, especially the content of photosynthetic pigments. The spectral light ratio (red:blue) proved to be the second factor affecting the studied properties. A higher red:blue ratio (70:30) was the best. Finally, it looks that the longest light period (10–14 h per day) did not play a significant role in establishing better photosynthetic performance and growth of cucumber seedlings. Therefore, it is necessary to perform molecular analyses and link them to morphological and biochemical traits to learn more about the mechanisms of the effect of LED light on seedling growth.

## Data Availability

All data available within the article.
